# Right ventricular function and anemia in heart failure with preserved ejection fraction

**DOI:** 10.3389/fcvm.2024.1424576

**Published:** 2024-11-18

**Authors:** Jia Wang, Jiahui Jiang, Xiang Li, Xilun Tan, Yanni Zhou, Ze Luo, Xuesen Wang, Xuezhu Zhao, Yiying Liu, Ming Wang, Chenhao Zhang

**Affiliations:** ^1^ Emergency Department, Wangjing Hospital of China Academy of Chinese Medical Sciences, Beijing, China; ^2^ Department of Cardiovascular Medicine, Chongqing Hospital of Traditional Chinese Medicine, Chongqing, China; ^3^ College of Traditional Chinese Medicine, Chongqing Medical University, Chongqing, China

**Keywords:** anemia, right ventricular dysfunction, right ventricular-pulmonary arterial coupling, heart failure, heart failure with preserved ejection fraction

## Abstract

**Background:**

Anemia is a common complication in patients with heart failure and is associated with left ventricular systolic dysfunction. However, its role in right ventricular (RV) function has not been evaluated.

**Methods:**

We retrospectively analyzed the electronic medical data of 1,014 Heart Failure with Preserved Ejection Fraction (HFpEF) patients to evaluate the relationship between anemia and RV dysfunction in patients with HFpEF and whether this relationship is influenced by classical risk factors such as smoking and hypertension.

**Results:**

The study showed that anemic patients were older and had significantly higher New York Heart Association functional class and tricuspid regurgitation (TR) than non-anemic patients. The level of hemoglobin (Hb) had a weak negative linear correlation with NT-pro-BNP (log-transform, *r* = 0.30, *P* < 0.0001) and a positively correlation with the tricuspid annular plane systolic excursion (TAPSE)/pulmonary arterial systolic pressure (PASP) ratio (*r* = 0.44, *P* < 0.0001). Multivariate linear regression analysis shows that the degree of anemia, atrial fibrillation, and TR were independently associated with the TAPSE/PASP ratio.

**Conclusion:**

Anemia in HFpEF is associated with RV dysfunction, and this relationship is not affected by classical risk factors, such as smoking, hypertension, and diabetes.

## Introduction

There is increasing recognition of the crucial role of the right ventricle (RV) in determining functional status and prognosis in multiple conditions (1–3). RV dysfunction (RVD) is mainly determined by the chief underlying process, including pressure overload, volume overload, or a primary myocardial process (4). Studies have indicated that RV pressure overload, most commonly secondary to pulmonary hypertension (PH), and myocardial insults from various etiologies lead to predominantly end-systolic and early diastolic flattening of the interventricular septum, and eventually, progressive RV dilation and dysfunction in heart failure reduced ejection fraction (HFrEF) (5, 6). However, right heart dysfunction due to afterload mismatch caused by impaired RV systolic function and PH is equally common in patients with HFpEF (7). More insights into the development of RVD in HFpEF may aid to our knowledge about this disease and ultimately contribute to better treatments to improve outcomes in these patients (8).

Identifying comorbidities that can influence the clinical course and response to treatment is very important in the management of heart failure (HF). In fact, a substantial percentage of hospitalizations in patients with HF are non-HF related and are not precipitated by a cardiac condition in more than half of cases (9–11). Anemia is a common comorbidity in patients with HF with an estimated approximately 30%–50% of patients with HF meeting World Health Organization (WHO) anemia thresholds [i.e., hemoglobin (Hb) <13.0 g/dl in men and <12.0 g/dl in women], which is associated with reduced ventricular free wall deformation and contractile reserve, and poor prognosis in patients with HF (12, 13). Anemia contributes to the morbidity, deterioration, and mortality of HF through several mechanisms, including increasing blood volume and cardiac workload, exacerbating myocardial hypoxia, and promoting cardiac enlargement (14). However, the vast majority of studies focused on the impact and role of anemia for left ventricular (LV) dysfunction, the investigation on association between anemia and RV impairment is very rare (15, 16).

Based on this literature review, we examined the effect of anemia on RV contractile performance and RV-pulmonary artery coupling in patients with HFpEF and explored the possible cause of RVD in this HF classification. These results may partly explain the mechanism by which anemia deteriorates cardiac function in HFpEF patients.

### Clinical significance

• The RV plays a crucial role in determining the functional status and prognosis of a variety of diseases. RVD caused by impaired RV systolic function and afterload mismatch caused by PH is common in HFpEF patients and is an important factor leading to poor prognosis of HFpEF.
• In the management of HF, identifying comorbidities that can influence the clinical course and response to treatment is crucial.
• Anemia is a common comorbidity in patients with HF and is associated with poor prognosis in patients with HF. However, the vast majority of studies have focused on the impact and role of anemia in LV dysfunction, and investigations on the association between anemia and RV impairment are rare.
• We explored the correlation between anemia and RV function in patients with HFpEF to elucidate the possible mechanism by which anemia affects the prognosis of HFpEF and provide a new target to optimize secondary prevention strategies for HF.

## Methods

**Figure 1:**
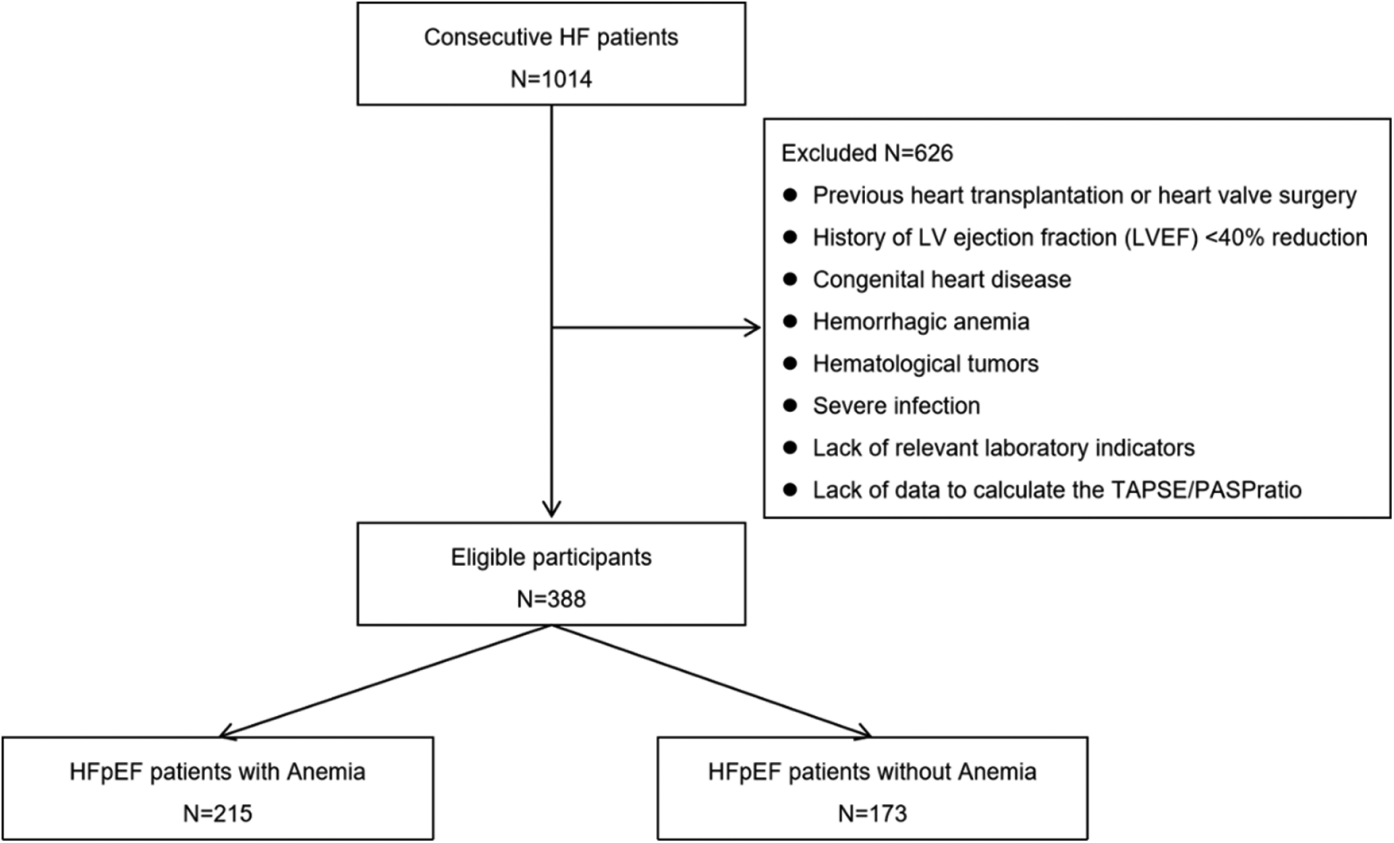
Flow of participants through the study.

We retrospectively analyzed consecutive 1,014 HF patients in the Department of Cardiovascular Medicine, Chongqing Hospital of Traditional Chinese Medicine from August 1, 2018 to October 1, 2020. The inclusion criteria were as follows: history, signs, symptoms, and treatment for HFpEF. Patients with HFpEF were selected according to the Chinese Guidelines for the diagnosis and treatment of Heart Failure 2018 (17). The diagnostic criteria for HFpEF are as follows: (1) presence of HF as defined by Framingham criteria, (2) left ventricular ejection fraction ≥50%, (3) NT-proBNP >125 pg/ml, and (3) transthoracic echocardiography meets at least one of the following diagnostic criteria (1) left ventricular hypertrophy and/or left atrial enlargement, and (2) ratio of early diastolic mitral inflow velocity (E) to early diastolic mitral annulus velocity (e') ≥ 13 and/or mean value of e' (interventricular septum and left ventricular lateral wall) <9 cm/s. Exclusion criteria were: previous heart transplantation or heart valve surgery, history of LV ejection fraction (LVEF) <40% reduction, congenital heart disease, acute or chronic hemorrhagic anemia, hematological tumors, severe infection, lack of relevant laboratory indicators, or lack of data to calculate the tricuspid annular plane systolic excursion (TAPSE)/pulmonary arterial systolic pressure (PASP) ratio. Thus, the final cohort included 388 HFpEF patients (Figure 1). Patients were divided into anemia and no anemia groups based on whether their Hb levels met the WHO anemia thresholds (i.e., male Hb <13.0 g/dl, female Hb <12.0 g/dl). The severity of anemia was classified according to *Practical Internal Medicine*, with mild anemia defined as an Hb level lower than normal but greater than 9.0 g/dl, moderate anemia defined as between 6.1 and 9.0 g/dl, and severe anemia defined as less than 6.1 g/dl (18). The study conformed to the principles outlined in the Declaration of Helsinki and was approved by the Institutional Review Board of Chongqing Traditional Chinese Medicine Hospital (Approval No. 2022-KY-KS-WJ) as posing minimal risk to patients, and was performed under a waiver of informed consent.

The following data were retrieved from the medical records: demographics, length of hospital stay, smoking history, comorbidities, New York Heart Association (NYHA) functional class, hemodynamics, and laboratory indicators. The admission value for all clinical measurements, and laboratory indicators was defined as the first value recorded from blood collection in the fasting state within 2 days of admission.

The echocardiogram data of Chongqing Hospital of Traditional Chinese Medicine were queried and the transthoracic echocardiogram data closest to the admission date were recorded. Transthoracic echocardiography was evaluated by GE-Vivid 7 Dimension (Vingmed, Horton, Norway). Each patient's LVEF was first assessed following the biplane Simpson's rule, and if not applicable, a two-dimensional or visually assessed LVEF was selected. TAPSE was measured by M-mode according to the American Society of Echocardiography, and the peak shift (mm) of the tricuspid annulus from end-diastolic to systolic was its value. RV systolic pressure was determined by measuring tricuspid regurgitation (TR) jet velocity according to the simplified Bernoulli equation and was considered to be approximately equal to PASP because the subject didn't have RV outflow tract obstruction. RV-pulmonary artery coupling was calculated by the ratio of TAPSE to echocardiography-derived PASP. The severity of TR was assessed semi-quantitatively based on the vena contracta width and density of the continuous wave signal (19).

Continuous and categorical variables are reported as mean ± SD and percentage, respectively. Comparisons between the groups (anemia vs. no anemia) were performed using unpaired *t*-test or Wilcoxon Rank-Sum Test for continuous variables and chi-square test for categorical variables. Linear regression analysis evaluated the relationship between hemoglobin levels and NT-pro-BNP (log-transformed) and the TAPSE/PASP ratio. Univariate and multivariate linear regression analyses were used to determine predictors of the TAPSE/SPAP index, and results were reported with standardized β-coefficients, *t*-values, and *P*-values. In the multiple linear regression analysis, we further examined the interaction of the degree of anemia with age and sex. A significance level of bilateral *P* < 0.05 was considered to be statistical meaning. Analysis of data utilizing IBM SPSS Statistics software, version 23.

## Results

The study population comprised 388 patients with a mean age of 75.7 ± 10.2 years, of whom 158 (40.7%) were men. Table 1 and Table 2 shows the patients' baseline characteristics in the study cohort according to the presence of anemia. Anemia was present in 215 patients (55.4%). The mean LVEF, TAPSE, PASP and TAPSE/PASP ratio were 62.0 ± 7.9%, 18.7 ± 5.2 mm, and 33.9 ± 15.0 mm/Hg, and 0.68 ± 0.40 respectively. Overall, Patients with anemia (*n* = 215) were older and had a significantly higher NYHA functional class and degree of TR than those without anemia. No substantial differences were observed in sex, smoking history, heart rate, systolic blood pressure, or the prevalence of diabetes or hypertension between patients with and without anemia. Moreover, patients with anemia had a longer hospital length of stay and displayed a history of atrial fibrillation more often. In terms of laboratory indicators, anemia patients showed lower values of alanine aminotransferase (ALT), triglyceride (TG), total cholesterol (TC), high-density lipoprotein (HDL), and low-density lipoprotein (LDL), and higher values of fibrinogen (FIB), C-reactive protein (CRP), and NT-pro-BNP. We found no differences in the aspartate aminotransferase (AST) levels (Table 2). Regarding echocardiographic parameters, patients with anemia exhibited higher left atrial diameter (LAD) values, lower TAPSE values, and a significantly decreased TAPSE/PASP ratio (*P* < 0.0001). Furthermore, PASP significantly increased (*P* < 0.0001), whereas LVEF was not significantly different (Table 2).

**Table 1:** Baseline demographic and clinical characteristics of the patients according to the presence of anemia.

	Total	Anemia	No anemia	*P*
Patients, *n*	388	215	173	
Sex,%male	158 (40.7)	92 (42.8)	66 (38.2)	0.3557
Age, years	75.7 ± 10.2	78.0 ± 8.9	73.1 ± 11.0	**<0**.**0001**
Body mass index, kg/m^2^	23.0 ± 2.5	22.9 ± 2.6	23.2 ± 2.5	0.3501
Hospital length of stay, day	9.9 ± 4.4	10.6 ± 4.8	9.0 ± 3.6	**0**.**0013**
Smoking history, *n* (%)	112 (28.9)	60 (27.9)	52 (30.1)	0.6426
Comorbidities				
Hypertension, *n* (%)	280 (72.2)	154 (71.6)	126 (72.8)	0.7927
Diabetes, *n* (%)	107 (27.6)	62 (28.8)	45 (26.0)	0.5364
Atrial fibrillation, *n* (%)	143 (36.9)	91 (42.3)	52 (30.1)	**0**.**0129**
NYHA functional class, *n* (%)				**0**.**0012**
I	2 (0.5)	0	2 (1.2)	
II	107 (27.6)	47 (21.9)	60 (34.7)	
III	214 (55.2)	125 (58.1)	89 (51.4)	
IV	65 (16.7)	43 (20.0)	22 (12.7)	
Haemodynamics				
Heart rate, bpm	85 ± 22	85 ± 23	85 ± 22	0.9267
Systolic blood pressure, mmHg	135 ± 23	134 ± 24	136 ± 22	0.3778
Diastolic blood pressure, mmHg	78 ± 16	75 ± 15	81 ± 17	**0**.**0007**

Bold values indicate *P* < 0.05.

Values are means ± SD.

NYHA, New York heart association.

**Table 2:** Baseline laboratory and echocardiographic characteristics of the patients according to the presence of anemia.

	Total	Anemia	No anemia	*P*
Laboratory indicators				
RBC	4.0 ± 0.9	3.7 ± 1.1	4.4 ± 0.4	**<0**.**0001**
HCT	36.9 ± 7.3	33.2 ± 4.5	41.4 ± 7.5	**<0**.**0001**
MCV	93.4 ± 14.4	92.3 ± 11.8	94.7 ± 17.0	0.7693
MCH	30.2 ± 3.6	29.5 ± 4.4	31.0 ± 2.0	**0**.**0002**
MCHC	324.6 ± 25.5	319.9 ± 27.7	330.4 ± 21.0	**<0**.**0001**
FIB	3.4 ± 2.4	3.5 ± 1.7	3.4 ± 3.0	**0**.**0090**
CRP	8.4 ± 13.5	10.2 ± 15.1	6.0 ± 10.8	**<0**.**0001**
ALT	22.7 ± 15.7	21.7 ± 14.8	24.0 ± 16.7	**0**.**0296**
AST	25.9 ± 16.1	25.8 ± 13.9	26.0 ± 18.5	0.5468
Cr	84.7 ± 40.2	91.9 ± 43.8	75.8 ± 33.3	**<0**.**0001**
TG	1.6 ± 1.0	1.5 ± 1.0	1.8 ± 1.1	**0**.**0001**
TC	4.2 ± 1.3	3.9 ± 1.1	4.5 ± 1.4	**<0**.**0001**
HDL	1.2 ± 0.5	1.2 ± 0.4	1.3 ± 0.5	**0**.**0016**
LDL	2.2 ± 0.9	2.0 ± 0.8	2.4 ± 0.9	**0**.**0001**
NT-pro-BNP	3467.4 ± 3535.5	4218.9 ± 3685.2	2533.5 ± 3105.7	**<0**.**0001**
TR, *n* (%)				**<0**.**0001**
Mild	250 (64.4)	105 (48.8)	145 (83.8)	
Moderate	61 (15.7)	48 (22.3)	13 (7.5)	
Severe	77 (19.9)	62 (28.9)	15 (8.7)	
Echocardiography				
LAD, mm	38.3 ± 8.0	39.6 ± 8.4	36.7 ± 7.1	**0**.**0006**
LVEF%	62.0 ± 7.9	61.8 ± 7.9	62.2 ± 7.9	0.6377
E/e'	15.5 ± 2.4	16.1 ± 2.6	14.8 ± 1.8	**<0**.**0001**
TAPSE, mm	18.7 ± 5.2	19.0 ± 18.0	19.2 ± 5.0	**0**.**0111**
PASP, mmHg	33.9 ± 15.0	41.0 ± 36.0	27.2 ± 12.1	**<0**.**0001**
TAPSE/PASP, mm/mmHg	0.68 ± 0.40	0.54 ± 0.28	0.85 ± 0.46	**<0**.**0001**

Bold values indicate *P* < 0.05.

Values are means ± SD.

RBC, red blood count; HCT, hematocrit; MCV, mean corpuscular volume; MCH, mean corpuscular hemoglobin; MCHC, mean corpuscular hemoglobin concentration; FIB, fibrinogen; CRP, C-reactive protein; ALT, alanine aminotransferase; AST, aspartate aminotransferase; Cr, creatinine; TG, triglyceride; TC, total cholesterol; HDL, high density lipoprotein; LDL, low density lipoprotein; NT-pro-BNP, NH2-terminal pro-brain-type natriuretic peptide; TR, tricuspid regurgitation; LAD, left atrial diameter; LVEF, left ventricular ejection fraction; E/e', early diastolic mitral inflow velocity/early diastolic mitral annulus velocity; TAPSE, tricuspid annular plane systolic excursion; PASP, pulmonary arterial systolic pressure.

**Figure 2:**
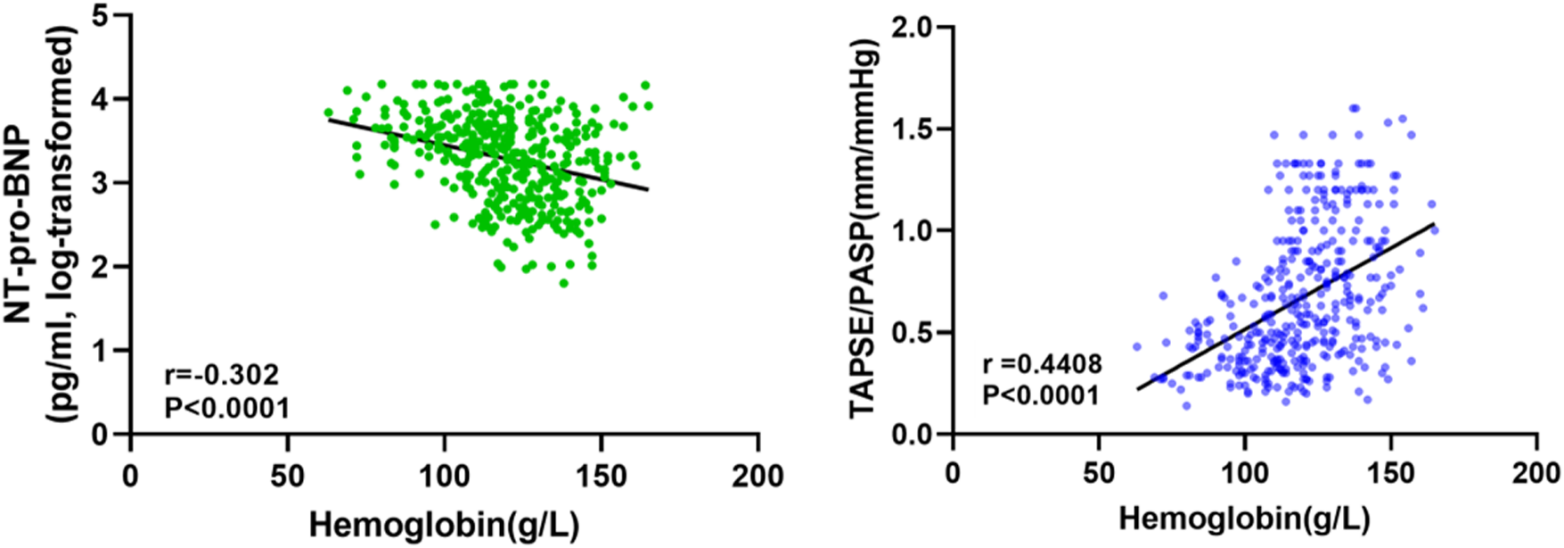
Relationship between serum NT-pro-BNP and hemoglobin concentration (left). Relationship between TAPSE/PASP ratio and hemoglobin concentration (right).

**Figure 3:**
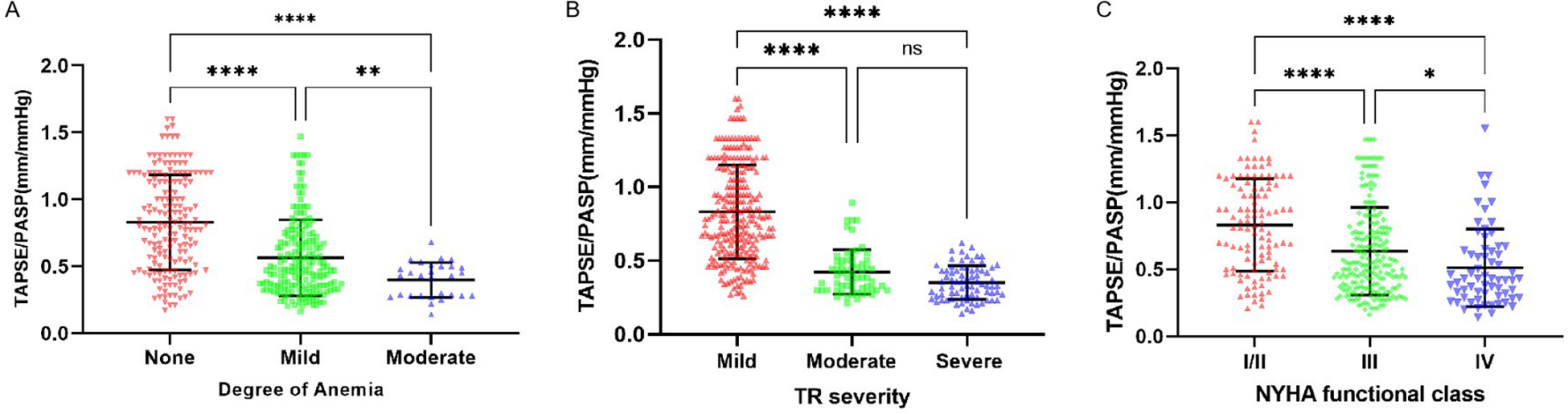
Relationship of the TAPSE/PASP ratio with degree of anemia, TR severity and NYHA functional class. Scatterplots show TAPSE/PASP ratio stratified by **(A)** Degree of anemia, **(B)** TR severity and **(C)** NYHA functional class. Line in panels A-C indicate median and interquartile range. *P < 0.05, ** *P* < 0.01, *** *P* < 0.001, ****P < 0.0001. NYHA, New York heart association; TR, tricuspid regurgitation; TAPSE, tricuspid annular plane systolic excursion; PASP, pulmonary arterial systolic pressure.

The relationship between the TAPSE/PASP ratio and Hb level, degree of TR, and NYHA functional class is shown in Figures 2, 3. Hb was weakly negatively linearly correlated with NT-pro-BNP (log-transformed, *r* = 0.30, *P* < 0.0001) and positively linearly correlated with TAPSE/PASP ratio (*r* = 0.44, *P* < 0.0001), as shown in Figure 2. As shown in Figure 3A, the TAPSE/PASP ratio was significantly lower in patients with moderate and mild anemia than in those without anemia (*P* < 0.0001). The TAPSE/PASP ratio was significantly lower in patients with moderate and severe TR than in patients with mild TR (*P* < 0.0001) (Figure 3B). In addition, there was an inverse relationship between the TAPSE/PASP ratio and NYHA functional class, and the TAPSE/PASP ratio decreased as the severity of the NYHA functional class increased (Figure 3C).

The important univariate correlations with the TAPSE/PASP ratio are shown in Table 3. The degree of TR was significantly negatively correlated with the TAPSE/PASP ratio in univariate analysis and remained a strong predictor of the TAPSE/SPAP ratio in the multivariate model (Table 3). In addition, the degree of anemia and atrial fibrillation were inversely associated with the TAPSE/PASP ratio in the univariate analysis and the remaining predictors of the TAPSE/SPAP ratio in the multivariate models (Table 3). The effect modification by sex or baseline age of the association between the degree of anemia and the TAPSE/SPAP ratio was not statistically significant (*P* > 0.05) based on the tests of interactions (data not shown).

**Table 3:** Univariate and multivariate covariates of TAPSE/PASP ratio in linear regression analyses.

	Univariate			Multivariate		
	*β*	*t*	*P*-value	*β*	*t*	*P*-value
Age, years	−0.21	−4.18	**<0**.**001**	−0.02	−1.04	0.297
Smoking history	−0.03	−0.55	0.585			
Hospital length of stay, day	−0.10	−1.98	0.049	0.02	0.92	0.358
Hypertension	−0.03	−0.55	0.585			
Atrial fibrillation	−0.45	−9.95	**<0**.**001**	−0.06	−2.40	**0**.**017**
Diabetes mellitus	0.13	2.56	0.011	0.04	1.67	0.095
NYHA functional class	−0.31	−6.32	**<0**.**001**	−0.03	−1.12	0.263
TR	−0.59	−14.35	**<0**.**001**	0.06	2.00	**0**.**046**
NT-pro-BNP	−0.38	−7.99	**<0**.**001**	−0.03	−1.04	0.299
RBC	0.28	5.75	**<0**.**001**	0.02	0.93	0.351
HCT	0.27	5.53	**<0**.**001**	−0.03	−1.05	0.295
Degree of Anemia	−0.43	−9.33	**<0**.**001**	−0.09	−2.04	**0**.**042**
Hb	0.44	9.55	**<0**.**001**	0.02	0.34	0.733
LAD, mm	−0.44	−9.69	**<0**.**001**	0.05	1.96	0.050
LVEF%	0.24	4.82	**<0**.**001**	0.06	2.63	**0**.**009**
TAPSE, mm	0.45	9.84	**<0**.**001**	0.32	13.69	**<0**.**001**
PASP, mmHg	−0.83	−29.80	**<0**.**001**	−0.76	−23.33	**<0**.**001**

Bold values indicate *P* < 0.05.

NYHA, New York heart association; TR, tricuspid regurgitation; NT-pro-BNP, NH2-terminal pro-brain-type natriuretic peptide; RBC, red blood count; HCT, hematocrit; Hb, hemoglobin; LAD, left atrial diameter; LVEF, left ventricular ejection fraction; TAPSE, tricuspid annular plane systolic excursion; PASP, pulmonary arterial systolic pressure.

## Discussion

In HF syndrome, the RV plays an increasingly recognized role in determining symptoms and outcomes in scenarios such as ischemic and nonischemic HF with either reduced or preserved ejection fraction (3, 8, 20). A large number of previous studies have focused on HFrEF, in which the mechanism of RVD is relatively clear. On the one hand, the left and right ventricles share myocardial fibers and interventricular septum, the left ventricle provides about 20%–40% of the contractile force to the RV, which further leads to the dysfunction of the RV when the left ventricle has systolic dysfunction (4). On the other hand, pulmonary hypertension (PH) leads to increased pulmonary circulatory resistance, and long-term after overload leads to RV remodeling and dysfunction (21). However, the patients with HFpEF rarely have myocardial systolic dysfunction and PH and there are also some clinical investigations which suggest that RVD may be present in up to one third of patients with HFpEF (22). At present, the main mechanism of RVD is caused by increased RV afterload and decreased pulmonary artery compliance due to elevated LA pressure, but HFpEF may also lead to RVD through a variety of other mechanisms (7). A deeper understanding of RVD in HFpEF could enhance our knowledge of the disease and potentially lead to improved treatments and outcomes for patients.

There is consensus that poor outcomes in patients with HF, including increased HF hospitalization rates and higher overall and cardiovascular mortality, are closely related to comorbidity in addition to dysfunction of the heart itself (23, 24). These comorbidities not only complicate the course of patients with HF but also has a substantial impact on the management of patients. Therefore, various clinical diseases that accompany HF are worthy of exploration for further optimization of secondary prevention strategies.

Anemia has been recognized as an important and common comorbidity in patients with HF, which is associated with adverse outcomes such as worse quality of life, increased hospitalization and mortality (25–27). The prevalence of anemia has been statistically ≈30% in stable patients and ≈50% in hospitalized patients with HF, with some reports describing higher prevalence rates (28, 29). In the studies of anemia and HF, most of them are discussed based on the effect and role of anemia on LV dysfunction. It is generally believed that in anemia, blood oxygen-carrying capacity decreases, tissue oxygen delivery is impaired, and cardiac output and blood flow compensatory increase improve hypoxia state, resulting in volume overload and flow overload (14, 16). Long-term flow/volume overload and cardiac work can induce eccentric LV hypertrophy (14). In addition, the increased sympathetic nerve activity of anemia can reduce renal blood flow, leading to water and sodium retention, increasing cardiac preload and further impairing LV function (30, 31). Studies have reported that anemia may contribute to HF by increasing LV filling pressure and left atrial enlargement, even in the absence of an underlying cardiovascular history (15). However, the correlation between anemia and RV function has been neglected and rarely reported.

Therefore, we further explored the correlation between anemia and RV function in patients with HFpEF to elucidate the possible mechanism by which anemia affects HFpEF prognosis. Considering that in the evaluation of RV function, the degree of coupling (or lack thereof) to the pulmonary circulation reflects the adequacy of RV contractility adaptation to afterload, RV-pulmonary artery coupling has become a comprehensive index to measure RV function (4). Although the comparison of Ea and Ees is the standard method of RV-pulmonary artery coupling assessment, its clinical application is limited by the invasive nature of right heart catheterization and the complexity of the calculation (32). Therefore, we estimate RV-pulmonary artery coupling by the ratio of TAPSE (as a surrogate of contractility) and PASP (as a surrogate of afterload), a simplified approach that has been proven to have a tight correlation with Ees/Ea and has been widely used clinically in recent years (33–35).

It is worth to note that Minana et al. explored the relationship between RV function and iron deficiency (ID) in patients with acute heart failure in their study, and found that transferrin saturation, a surrogate indicator of ID, was independently associated with lower TAPSE and TAPSE/PASP, but not with LVEF and PASP, confirming that ID was associated with RV dysfunction (36). However, because ID is only an important cause of anemia, it cannot explain all anemia phenomena. To the best of our knowledge, no papers have been published regarding the relationship between RV function and anemia in patients with HFpEF. Therefore, it is reasonable and interesting to investigate the relationship in patients with HFpEF. In this study, we analyzed the data of patients with almost all types of anemia, including ID, and observed that HFpEF patients with anemia had consistently higher PASP values and lower TAPSE values than those group without anemia. Previous studies have confirmed that the TASPE/PASP ratio has a significant ability to grade disease severity in patients with HF (37, 38). Our stud further confirms this conclusion, as shown in Figure 3B; the lower the TAPSE/PASP ratio, the more severe the degree of anemia and TR. Meanwhile, we observed higher NT-pro-BNP level in anemic patients, which may be due to decompensated HF and fluid overload in anemic patients. Furthermore, we also compared the coupling values with NYHA function, and as expected, RV-pulmonary artery coupling in HFpEF patients decreased with an increase in NYHA function class, consistent with the results of Guazzi et al. (39). Notably, our study shows that the degree of anemia is an independent predictor of the TAPSE/PASP ratio, suggesting that the degree of anemia shows a modest correlation with RV function, as indicated by the TAPSE/PAPS ratio, with RV function tending to decrease as anemia severity increases. Theoretically, the relationship between anemia and RVD may be multifaceted.

Iron deficiency (ID) plays an important role in this process (36). Although the cause of anemia in patients with HF is still not fully understood, the strongest evidence-based medical evidence for ID leading to anemia in HF is available (40). The mechanism of ID in patients with HF involves multiple pathways. Insufficient nutrient intake during HF and reduced gastrointestinal iron absorption due to intestinal wall edema caused by advanced mesenteric venous congestion (40, 41). In addition, due to intestinal wall edema causing changes in intestinal permeability and subsequent increase in bacterial concentration, IL-6 stimulates the production of the acute phase protein hepcidin in the liver while downregulating the expression of ferritin, inhibiting the duodenal absorption of iron, and preventing iron from being stored and released from the body (42–44). Moreover, patients with RVD have higher levels of venous congestion (36). Given that the liver is the main site of ferritin and transferrin synthesis, it is not difficult to speculate that congestive liver dysfunction in the course of HF leads to further exacerbation of ID. From animal models and clinical studies, there is increasing evidence that ID is associated with RVD (36, 45). As shown in animal model studies, ID can directly and rapidly promote pulmonary vascular remodeling, PH, and RV hypertrophy, and iron supplementation can reverse the pulmonary vascular remodeling caused by iron deficiency (45). Therefore, it is reasonable to speculate that ID in anemia may promote PH and right systolic dysfunction.

The second factor is the worsening of renal function. Kidney disease is an important cause of anemia in patients with HF through multiple mechanisms, such as impairment of erythropoietin production by mesenchymal duct pericytes (13, 40). A previous study by Napatt et al. showed a negative correlation between RV function and GFR and proposed the hypothesis that renal function worsened due to remodeling of the small pulmonary arteries in the long-term HFpEF state (46). In this study, HFpEF patients with anemia had higher Cr and PASP and lower RV-pulmonary artery coupling. This may be due to worsening RV structure/function leading to renal venous congestion and worsening chronic kidney disease, as suggested by Frank et al. (47). However, due to the cross-sectional nature of our study, we cannot prove causality. Renal impairment itself may also lead to worse prognosis in patients with anemic HFpEF.

Finally, AF may have an effect. Our research showed that the prevalence of AF was approximately 1.4 times higher in HFPEF patients with anemia than in those without anemia. As observed by Kotecha et al., when HFpEF is combined with AF, it results in decreased RV longitudinal contraction, RV dilation, and increased pulmonary artery pressure, leading to a decline in the overall function of the right heart (48, 49). A large number of previous studies have confirmed that anemia with AF is associated with an increased risk of cardiac events (50, 51). Therefore, it is not difficult to speculate that patients with anemia and HFpEF have a worse prognosis.

This study has several limitations. First, this was a cross-sectional, retrospective analysis; therefore, our conclusions must be interpreted as hypothesis generating, and causality cannot be inferred. Given that HF can also induce anemia through various mechanisms outlined above, particularly in the setting of volume overload, future evaluations of anemia and HF should exclude the possibility of acute volume overload. Additionally, since renal function plays a crucial role in the development of anemia, the lack of glomerular filtration rate data is a limitation of our study. Furthermore, because data on changes in anemia status were not obtained, it was not possible to assess the relationship between evolutionary changes in anemia parameters and RV function. Therefore, multicenter prospective studies are needed to investigate the impact of anemia with different etiologies on right ventricular function.

## Conclusion

Patients with HFpEF complicated by anemia have a marked decline in RV function irrespective of sex and age, and the degree of anemia is significantly negatively correlated with RV function. Furthermore, anemia, as an independent risk factor for RV function, is not associated with classic cardiovascular risk factors such as smoking, hypertension, and diabetes. Based on these results, we speculated that impaired RV function may be one of the important mechanisms by which anemia contributes to worsening heart failure.

## Data Availability

The datasets presented in this article are not readily available due to the nature of this research. The participants of this study did not agree for their data to be shared publicly, so supporting data is not available. Requests to access the datasets should be directed to Jia Wang, 3029197756@qq.com.

## References

[1] Xanthouli P Miazgowski J Benjamin N Gordjani O Egenlauf B Harutyunova S Prognostic meaning of right ventricular function and output reserve in patients with systemic sclerosis. Arthritis Res Ther. (2022) 24(1):173. 10.1186/s13075-022-02863-1 35864554 PMC9306074

[2] Meluzin J Spinarová L Hude P Krejcí J Dusek L Vítovec J Combined right ventricular systolic and diastolic dysfunction represents a strong determinant of poor prognosis in patients with symptomatic heart failure. Int J Cardiol. (2005) 105(2):164–73. 10.1016/j.ijcard.2004.12.031 16243108

[3] Santiago-Vacas E Lupón J Gavidia-Bovadilla G Gual-Capllonch F de Antonio M Domingo M Pulmonary hypertension and right ventricular dysfunction in heart failure: prognosis and 15-year prospective longitudinal trajectories in survivors. Eur J Heart Fail. (2020) 22(7):1214–25. 10.1002/ejhf.1862 32452102

[4] Sanz J Sánchez-Quintana D Bossone E Bogaard HJ Naeije R. Anatomy, function, and dysfunction of the right ventricle: JACC state-of-the-art review. J Am Coll Cardiol. (2019) 73(12):1463–82. 10.1016/j.jacc.2018.12.076 30922478

[5] Ghio S Raineri C Scelsi L Ašanin M Polovina M Seferovic P. Pulmonary hypertension and right ventricular remodeling in HFpEF and HFrEF. Heart Fail Rev. (2020) 25(1):85–91. 10.1007/s10741-019-09810-4 31197562

[6] Gomez-Arroyo J Santos-Martinez LE Aranda A Pulido T Beltran M Muñoz-Castellanos L Differences in right ventricular remodeling secondary to pressure overload in patients with pulmonary hypertension. Am J Respir Crit Care Med. (2014) 189(5):603–6. 10.1164/rccm.201309-1711LE 24579837

[7] Humbert M Kovacs G Hoeper MM Badagliacca R Berger RMF Brida M 2022 ESC/ERS guidelines for the diagnosis and treatment of pulmonary hypertension. Eur Heart J. (2022) 43(38):3618–731. 10.1093/eurheartj/ehac237 36017548

[8] Gorter TM van Veldhuisen DJ Bauersachs J Borlaug BA Celutkiene J Coats AJS Right heart dysfunction and failure in heart failure with preserved ejection fraction: mechanisms and management. Position statement on behalf of the heart failure association of the European society of cardiology. Eur J Heart Fail. (2018) 20(1):16–37. 10.1002/ejhf.1029 29044932

[9] Samuel NA Cuthbert JJ Brown OI Kazmi S Cleland JGF Rigby AS Relation between thyroid function and mortality in patients with chronic heart failure. Am J Cardiol. (2021) 139:57–63. 10.1016/j.amjcard.2020.10.034 33115640

[10] Kumowski N Marx N Schütt K. Treating heart failure in patients with diabetes: the view of the cardiologist. Diabetes Res Clin Pract. (2021) 176:108852. 10.1016/j.diabres.2021.108852 33957143

[11] Carlisle MA Fudim M DeVore AD Piccini JP. Heart failure and atrial fibrillation, like fire and fury. JACC Heart Fail. (2019) 7(6):447–56. 10.1016/j.jchf.2019.03.005 31146871

[12] Ambrosy AP Gurwitz JH Tabada GH Artz A Schrier S Rao SV Incident anaemia in older adults with heart failure: rate, aetiology, and association with outcomes. Eur Heart J Qual Care Clin Outcomes. (2019) 5(4):361–9. 10.1093/ehjqcco/qcz010 30847487 PMC6775859

[13] Anand IS. Heart failure and anemia: mechanisms and pathophysiology. Heart Fail Rev. (2008) 13(4):379–86. 10.1007/s10741-008-9088-8 18236152

[14] Metivier F Marchais SJ Guerin AP Pannier B London GM. Pathophysiology of anaemia: focus on the heart and blood vessels. Nephrol Dial Transplant. (2000) 15(3):14–8. 10.1093/oxfordjournals.ndt.a027970 11032352

[15] Cho IJ Mun YC Kwon KH Shin GJ. Effect of anemia correction on left ventricular structure and filling pressure in anemic patients without overt heart disease. Korean J Intern Med. (2014) 29(4):445–53. 10.3904/kjim.2014.29.4.445 25045292 PMC4101591

[16] McCullough PA Barnard D Clare R Ellis SJ Fleg JL Fonarow GC Anemia and associated clinical outcomes in patients with heart failure due to reduced left ventricular systolic function. Clin Cardiol. (2013) 36(10):611–20. 10.1002/clc.22181 23929781 PMC4008125

[17] Heart Failure Group of Chinese Society of Cardiology of Chinese Medical Association CHFAoCMDA, Editorial Board of Chinese Journal of Cardiology. Chinese Guidelines for the diagnosis and treatment of heart failure 2018. Zhonghua Xin Xue Guan Bing Za Zhi. (2018) 46(10):760–89. 10.3760/cma.j.issn.0253-3758.2018.10.004 30369168

[18] Chan HZ Lin GW. Practical Internal Medicine. 14th ed. Beijing: People’s Medical Publishing House (2013).

[19] Lancellotti P Tribouilloy C Hagendorff A Popescu BA Edvardsen T Pierard LA Recommendations for the echocardiographic assessment of native valvular regurgitation: an executive summary from the European association of cardiovascular imaging. Eur Heart J Cardiovasc Imaging. (2013) 14(7):611–44. 10.1093/ehjci/jet105 23733442

[20] Katz DH Burns JA Aguilar FG Beussink L Shah SJ. Albuminuria is independently associated with cardiac remodeling, abnormal right and left ventricular function, and worse outcomes in heart failure with preserved ejection fraction. JACC Heart Fail. (2014) 2(6):586–96. 10.1016/j.jchf.2014.05.016 25282032 PMC4256131

[21] Damy T Goode KM Kallvikbacka-Bennett A Lewinter C Hobkirk J Nikitin NP Determinants and prognostic value of pulmonary arterial pressure in patients with chronic heart failure. Eur Heart J. (2010) 31(18):2280–90. 10.1093/eurheartj/ehq245 20693169

[22] Zakeri R Mohammed SF. Epidemiology of right ventricular dysfunction in heart failure with preserved ejection fraction. Curr Heart Fail Rep. (2015) 12(5):295–301. 10.1007/s11897-015-0267-3 26338372

[23] Paolillo S Scardovi AB Campodonico J. Role of comorbidities in heart failure prognosis part I: anaemia, iron deficiency, diabetes, atrial fibrillation. Eur J Prev Cardiol. (2020) 27(2):27–34. 10.1177/2047487320960288 33238738 PMC7691628

[24] van Deursen VM Urso R Laroche C Damman K Dahlström U Tavazzi L Co-morbidities in patients with heart failure: an analysis of the European heart failure pilot survey. Eur J Heart Fail. (2014) 16(1):103–11. 10.1002/ejhf.30 24453099

[25] Tang YD Katz SD. The prevalence of anemia in chronic heart failure and its impact on the clinical outcomes. Heart Fail Rev. (2008) 13(4):387–92. 10.1007/s10741-008-9089-7 18246424

[26] Ezekowitz JA McAlister FA Armstrong PW. Anemia is common in heart failure and is associated with poor outcomes: insights from a cohort of 12 065 patients with new-onset heart failure. Circulation. (2003) 107(2):223–5. 10.1161/01.CIR.0000052622.51963.FC 12538418

[27] Xia H Shen H Cha W Lu Q. The prognostic significance of Anemia in patients with heart failure: a meta-analysis of studies from the last decade. Front Cardiovasc Med. (2021) 8:632318. 10.3389/fcvm.2021.632318 34055927 PMC8155282

[28] Anand IS Gupta P. Anemia and iron deficiency in heart failure: current concepts and emerging therapies. Circulation. (2018) 138(1):80–98. 10.1161/CIRCULATIONAHA.118.030099 29967232

[29] Sharma YP Kaur N Kasinadhuni G Batta A Chhabra P Verma S Anemia in heart failure: still an unsolved enigma. Egypt Heart J. (2021) 73(1):75. 10.1186/s43044-021-00200-6 34453627 PMC8403217

[30] McCullough PA Lepor NE. The deadly triangle of anemia, renal insufficiency, and cardiovascular disease: implications for prognosis and treatment. Rev Cardiovasc Med. (2005) 6(1):1–10. 10.1016/j.carrev.2005.06.002 15741920

[31] Anand IS Chandrashekhar Y Ferrari R Poole-Wilson PA Harris PC. Pathogenesis of oedema in chronic severe anaemia: studies of body water and sodium, renal function, haemodynamic variables, and plasma hormones. Br Heart J. (1993) 70(4):357–62. 10.1136/hrt.70.4.357 8217445 PMC1025332

[32] Nie L Li J Zhang S Dong Y Xu M Yan M Correlation between right ventricular-pulmonary artery coupling and the prognosis of patients with pulmonary arterial hypertension. Medicine. (2019) 98(40):e17369. 10.1097/MD.0000000000017369 31577738 PMC6783205

[33] Santas E Palau P Guazzi M de la Espriella R Miñana G Sanchis J Usefulness of right ventricular to pulmonary circulation coupling as an indicator of risk for recurrent admissions in heart failure with preserved ejection fraction. Am J Cardiol. (2019) 124(4):567–72. 10.1016/j.amjcard.2019.05.024 31204033

[34] Saeed S Smith J Grigoryan K Lysne V Rajani R Chambers JB. The tricuspid annular plane systolic excursion to systolic pulmonary artery pressure index: association with all-cause mortality in patients with moderate or severe tricuspid regurgitation. Int J Cardiol. (2020) 317:176–80. 10.1016/j.ijcard.2020.05.093 32512064

[35] Tello K Axmann J Ghofrani HA Naeije R Narcin N Rieth A Relevance of the TAPSE/PASP ratio in pulmonary arterial hypertension. Int J Cardiol. (2018) 266:229–35. 10.1016/j.ijcard.2018.01.053 29887454

[36] Miñana G Santas E de la Espriella R Núñez E Lorenzo M Núñez G Right ventricular function and iron deficiency in acute heart failure. Eur Heart J Acute Cardiovasc Care. (2021) 10(4):406–14. 10.1093/ehjacc/zuaa028 33620455

[37] Santas E De la Espriella R Chorro FJ Palau P Miñana G Heredia R Right ventricular dysfunction staging system for mortality risk stratification in heart failure with preserved ejection fraction. J Clin Med. (2020) 9(3):831–44. 10.3390/jcm9030831 32197527 PMC7141269

[38] Nakagawa A Yasumura Y Yoshida C Okumura T Tateishi J Yoshida J Prognostic importance of right ventricular-vascular uncoupling in acute decompensated heart failure with preserved ejection fraction. Circ Cardiovasc Imaging. (2020) 13(11):e011430. 10.1161/CIRCIMAGING.120.011430 33198494

[39] Guazzi M Bandera F Pelissero G Castelvecchio S Menicanti L Ghio S Tricuspid annular plane systolic excursion and pulmonary arterial systolic pressure relationship in heart failure: an index of right ventricular contractile function and prognosis. Am J Physiol Heart Circ Physiol. (2013) 305(9):H1373–81. 10.1152/ajpheart.00157.2013 23997100

[40] Sîrbu O Floria M Dascalita P Stoica A Adascalitei P Sorodoc V Anemia in heart failure—from guidelines to controversies and challenges. Anatol J Cardiol. (2018) 20(1):52–9. 10.14744/AnatolJCardiol.2018.08634 29952364 PMC6237795

[41] Vest AR Chan M Deswal A Givertz MM Lekavich C Lennie T Nutrition, obesity, and cachexia in patients with heart failure: a consensus statement from the heart failure society of america scientific statements committee. J Card Fail. (2019) 25(5):380–400. 10.1016/j.cardfail.2019.03.007 30877038

[42] Weiss G Ganz T Goodnough LT. Anemia of inflammation. Blood. (2019) 133(1):40–50. 10.1182/blood-2018-06-856500 30401705 PMC6536698

[43] Muckenthaler MU Rivella S Hentze MW Galy B. A red carpet for iron metabolism. Cell. (2017) 168(3):344–61. 10.1016/j.cell.2016.12.034 28129536 PMC5706455

[44] Odeh M Sabo E Oliven A. Circulating levels of tumor necrosis factor-alpha correlate positively with severity of peripheral oedema in patients with right heart failure. Eur J Heart Fail. (2006) 8(2):141–6. 10.1016/j.ejheart.2005.05.010 16112904

[45] Cotroneo E Ashek A Wang L Wharton J Dubois O Bozorgi S Iron homeostasis and pulmonary hypertension: iron deficiency leads to pulmonary vascular remodeling in the rat. Circ Res. (2015) 116(10):1680–90. 10.1161/CIRCRESAHA.116.305265 25767292

[46] Kanjanahattakij N Sirinvaravong N Aguilar F Agrawal A Krishnamoorthy P Gupta S. High right ventricular stroke work Index is associated with worse kidney function in patients with heart failure with preserved ejection fraction. Cardiorenal Med. (2018) 8(2):123–9. 10.1159/000486629 29617005 PMC5968285

[47] Dini FL Demmer R Simoniuc A Donati F Marzilli M Colombo PC. Right ventricular function is a critical determinant of chronic kidney disease and prognosis in patients with chronic systolic heart failure. J Am Coll Cardiol. (2011) 57(14):E354-E. 10.1016/S0735-1097(11)60354-9

[48] Kotecha D Lam CS Van Veldhuisen DJ Van Gelder IC Voors AA Rienstra M. Heart failure with preserved ejection fraction and atrial fibrillation: vicious twins. J Am Coll Cardiol. (2016) 68(20):2217–28. 10.1016/j.jacc.2016.08.048 27855811

[49] Gorter TM van Melle JP Rienstra M Borlaug BA Hummel YM van Gelder IC Right heart dysfunction in heart failure with preserved ejection fraction: the impact of atrial fibrillation. J Card Fail. (2018) 24(3):177–85. 10.1016/j.cardfail.2017.11.005 29197548

[50] Lee WH Hsu PC Chu CY Lee HH Lee MK Lee CS Anemia as an independent predictor of adverse cardiac outcomes in patients with atrial fibrillation. Int J Med Sci. (2015) 12(8):618–24. 10.7150/ijms.11924 26283880 PMC4532968

[51] An Y Ogawa H Esato M Ishii M Iguchi M Masunaga N Cardiovascular events and mortality in patients with atrial fibrillation and anemia (from the fushimi AF registry). Am J Cardiol. (2020) 134:74–82. 10.1016/j.amjcard.2020.08.009 32900468

